# Identification of Drug–Cancer Associations: A Nationwide Screening Study

**DOI:** 10.1158/2767-9764.CRC-22-0026

**Published:** 2022-06-29

**Authors:** Kasper Bruun Kristensen, Søren Friis, Lars Christian Lund, Jesper Hallas, Chris R. Cardwell, Bettina K. Andreassen, Laurel A. Habel, Anton Pottegård

**Affiliations:** 1 Clinical Pharmacology, Pharmacy and Environmental Medicine, Department of Public Health, University of Southern Denmark, Odense, Denmark.; 2 Danish Cancer Society Research Center, Danish Cancer Society, Copenhagen, Denmark.; 3 Centre for Public Health, Queen's University Belfast, Belfast, United Kingdom.; 4 Department of Research, Cancer Registry of Norway, Oslo, Norway.; 5 Division of Research, Kaiser Permanente Northern California, Oakland, California.

## Abstract

The main tool in drug safety monitoring, spontaneous reporting of adverse effects, is unlikely to detect delayed adverse drug effects including cancer. Hypothesis-free screening studies based on administrative data could improve ongoing drug safety monitoring. Using Danish health registries, we conducted a series of case–control studies by identifying individuals with incident cancer in Denmark from 2001 to 2018, matching each case with 10 population controls on age, sex, and calendar time. ORs were estimated using conditional logistic regression accounting for matching factors, educational level, and selected comorbidities. A total of 13,577 drug–cancer associations were examined for individual drugs and 8,996 for drug classes. We reviewed 274 drug–cancer pairs where an association with high use and a cumulative dose–response pattern was present. We classified 65 associations as not readily attributable to bias of which 20 were established as carcinogens by the International Agency for Research on Cancer and the remaining 45 associations may warrant further study. The screening program identified drugs with known carcinogenic effects and highlighted a number of drugs that were not established as carcinogens and warrant further study. The effect estimates in this study should be interpreted cautiously and will need confirmation targeted epidemiologic and translational studies.

**Significance:**

This study provides a screening tool for drug carcinogenicity aimed at hypothesis generation and explorative purposes. As such, the study may help to identify drugs with unknown carcinogenic effects and, ultimately, improve drug safety as part of the ongoing safety monitoring of drugs.

## Introduction

When new drugs are approved, safety monitoring for adverse effects (pharma-covigilance) plays a central role. However, at the time of market entry of a given drug, there is limited evidence regarding late adverse effects such as cancer. Since cancer usually occurs years after the initial exposure, is a rare event, and may occur after the drug has been discontinued, drug-related cancers are rarely detected by spontaneous reporting of adverse events or in premarketing randomized clinical trials. Observational studies have been instrumental to identify carcinogenic effects of drugs, for example, phenacetin and upper urinary tract cancers (1). A few drugs, such as phenacetin, have been classified as carcinogenic by the International Agency for Research on Cancer (IARC) while a larger number of drugs are classified as probably or possibly carcinogenic (2).

Denmark has a long tradition for keeping administrative and health registries that are well-suited for population-based observational studies (3). The Danish National Prescription Registry has collected information on all prescriptions filled at community pharmacies since 1995 and The Danish Cancer Registry holds data on incident cancers since 1943 (4, 5). These data provide a unique opportunity to establish a hypothesis-free screening program for potential carcinogenic effects of drugs. This program was established in 2016 and included 278,485 individuals with incident cancer in Denmark (6). The program identified an association between hydrochlorothiazide and nonmelanoma skin cancer (7, 8), a finding that was replicated in other populations (9, 10), resulted in safety updates from regulatory agencies (11), and changed prescribing patterns of hydrochlorothiazide (12). This study was conducted as part of the ongoing surveillance of drug carcinogenicity and provides results for incident cancers in Denmark from 2001 to 2018 using updated methodology and presenting all examined drug–cancer associations via an interactive online tool.

## Materials and Methods

### Data Sources

The screening tool was based on Danish health and administrative registries. All residents in Denmark are assigned a unique personal identification number and have access to universal tax-supported health care enabling population-based studies on all Danish residents (approximately 5.8 million; ref. 3). The Danish Civil Registration system records data on vital status and migration to and from Denmark and was used to define the source population of the case–control study (13). Cancer outcomes were identified from the Danish Cancer Registry (5). We used this registry to identify incident cancers coded according to the International Classification of Diseases version 10 (ICD-10; ref. 14). Approximately 90% of cancers in the Danish Cancer Registry are histologically verified and classified using the ICD for Oncology version 3 (ICD-O-3; ref. 15). The Danish National Prescription registry holds data on all filled prescriptions at community pharmacies in Denmark since 1995, including the name of the drug, the dispensed volume and strength and the date of dispensing (4). Drug substances are classified according to the Anatomical Therapeutic Chemical (ATC) index (16). The Danish National Patient Register was used to obtain information on hospital diagnoses for confounder adjustment using information on all in- and outpatient as well as emergency department diagnoses in Denmark since 1995 (17). The Danish Education Registries administered by Statistics Denmark were used to obtain information on highest achieved educational level (18).

### Cancer Outcomes

The case-defining cancers included the most common cancers except nonmelanoma skin cancer and were classified according to affected site using ICD-10 codes and according to histologic subtype using ICD-O-3 morphology codes (Supplementary Table S1). The histologic classification was based on manual review of all morphology codes for each cancer and was largely guided by the current WHO Classification of Tumors series (19); however, because the morphologic classification of cancers changed during the study period, commonly used clinical definitions were also taken into account. Cancers that were not histologically verified were excluded except tumors of the central nervous system and hematologic malignancies. The main outcome of interest was cancer defined by site and histologic subtype, for example, small-cell carcinoma of the lung. As secondary outcomes, we included cancers defined by the affected site, for example, lung cancer.

### Study Population

Cases were all Danish residents with incident cancer during January 1, 2001 to December 31, 2018. We excluded individuals with previous (1978–) cancer, except nonmelanoma skin cancer, to increase the specificity of the included cancer outcomes as primary, incident cancers and because oncologic therapy may increase susceptibility to other malignancies. Because drug use and cancer incidence are limited among children and adolescents, cases below 18 years of age were excluded. To ensure at least 10 years of follow-up prior to the cancer diagnosis, cases who migrated to or from Denmark during the 10 years before the index date were excluded. Controls were selected using risk-set sampling by matching up to 10 controls to each case on sex and age. Controls were assigned an index date corresponding to the date of the cancer diagnosis of their matched case and were alive, residents in Denmark and at risk of their first cancer at the index date. The same exclusion criteria were applied as for cases, that is, individuals with any cancer diagnosis except nonmelanoma skin cancer before the index date, age below 18 years at the index date, or migrations during the 10 years prior to the index date were not eligible as controls. Cases were eligible for sampling as controls before their cancer diagnosis and each individual could be sampled more than once. With this sampling scheme, the ORs are estimates of the incidence rate ratio from a cohort study of the entire source population (20).

### Classification of Drug Exposures

In the main analyses, we classified drugs according to the ATC index on the fifth level (e.g., C07AB02 metoprolol). Secondarily, we examined drug classes according to the fourth ATC level (e.g., C07AB selective beta-blockers). The 2020 WHO ATC classification was used (16). Drug exposure was assessed for cases and controls from 1995 until two years before the index date. We disregarded drug use during the two years before the index date because recent exposure is unlikely to cause cancer and to reduce protopathic bias (reverse causation) and surveillance bias (21).

### Covariates

We adjusted for highest achieved education as a proxy of socioeconomic status (none or basic education; high school or vocational training; higher education; unknown) and the Charlson Comorbidity Index summary score, CCI (ref. 22; Supplementary Table S2). The Charlson comorbidity entities any malignancy including leukemia and lymphoma and metastatic solid tumor were not included because study subjects with cancer prior to the index date were excluded. Similar to the exposure assessment, covariates were assessed until 2 years before the index-date.

### Statistical Analyses

Drug exposure was modeled by number of filled prescriptions categorized as nonuse (0 prescriptions), low use (1–2 prescriptions), intermediate use (3–7 prescriptions), and high use (8 or more prescriptions). The main exposure of interest was 8 or more prescription fills and we did not impose additional requirements for time intervals between prescriptions or the prescriptions being consecutive. The cutoff for high use was chosen because drugs for chronic conditions are typically dispensed in 3-month intervals in Denmark whereby 8 prescription fills are assumed to correspond to approximately 2 years of treatment for drugs used to treat chronic conditions. The threshold of 8 prescriptions were in line with previous similar works and chosen because a certain cumulative dose is usually needed before cancer development is plausibly affected (23, 24). We only analyzed drug–cancer pairs where the number of cases exposed to high use was 25 or above. Considering that the number of exposed cases is the limiting factor for statistical precision, a bottleneck analysis can be carried out where the theoretical optimum achievable statistical precision of a null result (OR = 1) with 25 exposed cases would be estimated as a 95% confidence interval ranging from 0.7 to 1.5 (25).

We estimated ORs for high use compared with nonuse using conditional logistic regression with cumulative number of filled prescriptions (categorical), CCI (numeric), and education (categorical) as independent variables. High use compared with nonuse was the main exposure; however, we also estimated ORs for low use compared with nonuse.

To examine cumulative dose–response patterns, we fitted a conditional logistic regression model including the cumulative number of filled prescriptions as an independent, continuous variable with an indicator term for ever-use versus never-use. The cumulative number of filled prescriptions was log_2_-transformed, and with the indicator term for ever-use included, the model estimated the OR associated with each doubling of the cumulative number of filled prescriptions among ever-users (Supplementary Methods S3). In these analyses, we included educational level and CCI as covariates as in the other analyses.

Statistical analyses were conducted in Stata version 17.0 (StataCorp, RRID:SCR_012763) and the online tool was constructed using the DT and flexdashboard packages in R version 4.1.1 (R Foundation for Statistical Computing, RRID:SCR_001905; refs. 26, 27).

### Evaluation of Drug–Cancer Associations

To identify drug–cancer associations for manual review from the main analyses, we applied thresholds related to the strength of association with high use and the presence of a cumulative dose–response relationship. We identified associations where the lower limit of the 95% CI for high use was above 1.25 whereafter associations with a 95% CI lower limit above 1 for each doubling of cumulative dose were kept. These associations were manually reviewed by three authors (K.B. Kristensen, L.C. Lund, A. Pottegård) and classified in three groups guided by known risk factors for the cancer in question and considerations regarding indications of the drug in question: (i) associations that were likely explained by bias; (ii) drugs with limited systemic absorption unlikely to affect cancer development; and (iii) drug–cancer associations not readily attributable to bias. Each drug–cancer association was independently reviewed by the three authors whereafter disagreements were solved by consensus. Available evidence was identified using existing reference works by the International Agency for Research on Cancer (2) and by searching MEDLINE using text words for the drug and specific cancer type in question. The drug–cancer associations classified in group (iii) were evaluated as to whether they were established carcinogens by the IARC and if so, if they were established as carcinogens with sufficient evidence in humans [group (i)], or carcinogens with limited evidence in humans [group (ii); ref. 2]. The drug–cancer associations classified in group (iii), were compared with two recent drug–cancer screening studies from Norway and Scotland (23, 24). In the Norwegian study, ATC codes were truncated to the 4th level and the effect estimates were for 8 or more prescriptions compared with never-use and adjusted for comorbid conditions, use of other drugs, parity for females, and county of residence (23). In the Scottish study, the effect estimates were for 6 or more prescriptions compared with less than six prescriptions and adjusted for comorbid conditions and specific risk factors for the cancer outcome of interest (24). The exact cancer outcomes and drug exposures for the Norwegian and Scottish estimates are shown in the online tool.

### Data Availability

Because of data protection regulations and patient privacy, individual level data as used in this study cannot be shared by the authors. Data access can be granted to university based Danish scientific organizations after application to a third party, Statistics Denmark (https://www.dst.dk/en/kontakt).

### Ethics Approval

The study was approved by the University of Southern Denmark (reference no 10.522). Ethical approval is not required for register-based studies in Denmark.

## Results

### Study Population and Drug–Cancer Associations

We identified 456,828 individuals with incident cancer (cases) and matched them to 4,568,262 controls (Table 1). The most common cancers were colorectal adenocarcinoma (*n* = 63,992), prostatic adenocarcinoma (*n* = 61,024), ductal carcinoma of the breast (*n* = 54,238), malignant melanoma of the skin (*n* = 29,676), and lung and trachea adenocarcinoma (*n* = 25,944). The total number of examined drug–cancer associations was 13,577 for individual drugs and 8,996 for drug classes (Supplementary Table S4). All examined associations are available in the online tool (pharmacoepi.sdu.dk/cancerscreening). In Table 2, the drug–cancer pairs with the 10 highest ORs for high use and the 10 highest ORs for each doubling of cumulative dose in users are shown. The strongest associations for high use were seen for antibiotics used to treat urinary tract infections (pivmecillinam and nitrofurantoin) and squamous cell carcinoma of the bladder. The benzodiazepine drug chlordiazepoxide accounted for 3 of the 10 highest ORs being strongly associated with squamous cell carcinoma of the larynx and hypopharynx, oral cavity and oropharynx, and esophagus—cancers that are mainly caused by smoking and alcohol (28). The highest ORs for each doubling of cumulative dose in users were for antibiotics used to treat urinary tract infections (pivmecillinam and nitrofurantoin) and risk of squamous cell carcinoma of the bladder.

**TABLE 1:** Basic characteristics for cases and controls for the ten most common histologic subtypes of cancer

					Highest achieved education			
	Cases/ controls, *n*	Age, M (p^25^–p^75^)	Male sex, *n* (%)	CCI, mean (SD)	None or basic	High school or vocational	Higher education	Unknown
Colorectal (adenocarcinoma)								
Cases	63,922	71 (63–79)	34,591 (54)	0.42 (0.86)	24,539 (38)	25,302 (40)	9,546 (15)	4,535 (7)
Controls	639,217		345,910 (54)	0.42 (0.86)	244,839 (38)	246,690 (39)	104,851 (16)	42,837 (7)
Prostate (adenocarcinoma)								
Cases	61,024	70 (65–76)	61,024 (100)	0.40 (0.81)	18,408 (30)	27,961 (46)	11,991 (20)	2,664 (4)
Controls	610,240		610,240 (100)	0.48 (0.92)	203,799 (33)	273,914 (45)	104,580 (17)	27,947 (5)
Breast, female (ductal carcinoma)								
Cases	54,238	62 (53–70)	–	0.25 (0.64)	18,207 (34)	21,405 (39)	12,606 (23)	2,020 (4)
Controls	542,380		–	0.25 (0.64)	197,973 (37)	206,766 (38)	117,445 (22)	20,196 (4)
Skin (melanoma)								
Cases	29,676	60 (46–71)	13,566 (46)	0.26 (0.70)	7,252 (24)	13,785 (46)	7,682 (26)	957 (3)
Controls	296,760		135,660 (46)	0.29 (0.73)	92,486 (31)	130,344 (44)	62,751 (21)	11,179 (4)
Lung and trachea (adenocarcinoma)								
Cases	25,944	68 (61–75)	11,633 (45)	0.54 (0.95)	11,631 (45)	10,367 (40)	3,075 (12)	871 (3)
Controls	259,440		116,330 (45)	0.37 (0.81)	96,483 (37)	107,094 (41)	48,765 (19)	7,098 (3)
Lymphoma, non-Hodgkin								
Cases	16,947	68 (59–77)	9,426 (56)	0.42 (0.87)	6,213 (37)	6,854 (40)	2,966 (18)	914 (5)
Controls	169,464		94,260 (56)	0.38 (0.82)	61,242 (36)	69,534 (41)	29,991 (18)	8,697 (5)
Lung and trachea (squamous cell carcinoma)								
Cases	12,838	71 (65–77)	8,483 (66)	0.71 (1.08)	6,538 (51)	4,645 (36)	935 (7)	720 (6)
Controls	128,380		84,830 (66)	0.44 (0.88)	51,098 (40)	51,900 (40)	20,660 (16)	4,722 (4)
Bladder (urothelial carcinoma)								
Cases	12,126	73 (66–80)	9,065 (75)	0.55 (0.96)	4,900 (40)	4,866 (40)	1,398 (12)	962 (8)
Controls	121,260		90,650 (75)	0.47 (0.91)	45,754 (38)	46,974 (39)	18,824 (16)	9,708 (8)
Lung and trachea (small-cell carcinoma)								
Cases	9,612	69 (62–75)	4,890 (51)	0.60 (1.00)	4,835 (50)	3,541 (37)	856 (9)	380 (4)
Controls	96,120		48,900 (51)	0.37 (0.80)	37,975 (40)	38,446 (40)	16,853 (18)	2,846 (3)
Lung and trachea (carcinoma, other and unspecified)								
Cases	9,436	70 (62–76)	5,111 (54)	0.61 (1.02)	4,603 (49)	3,521 (37)	840 (9)	472 (5)
Controls	94,360		51,110 (54)	0.38 (0.82)	37,716 (40)	37,395 (40)	15,534 (16)	3,715 (4)

Abbreviations: CCI; Charlson Comorbidity Index; M (p^25^–p^75^), Median (25th percentile – 75th percentile).

**TABLE 2:** Number of cases with high use (8 or more prescriptions), ORs for high use, ORs for each doubling of cumulative dose in users, and ORs for low use (1–2 prescriptions) for selected drug–cancer pairs

	ATC	Drug name	Exposed cases, n	OR high use (95% CI)	OR cumulative dose–response (95% CI)	OR low use (95% CI)
**10 highest ORs for high use**						
Bladder (squamous cell carcinoma)	J01CA08	Pivmecillinam	40	15.35 (9.40–25.06)	1.68 (1.47–1.92)	2.43 (1.81–3.25)
Bladder (squamous cell carcinoma)	J01XE01	Nitrofurantoin	30	13.58 (7.85–23.51)	1.46 (1.25–1.71)	3.87 (2.82–5.30)
Liver (Hepatocellular carcinoma)	N07BC02	Methadone	62	12.02 (7.76–18.63)	1.43 (1.26–1.62)	1.69 (0.74–3.85)
Larynx and hypopharynx (squamous cell carcinoma)	N05BA02	Chlordiazepoxide	122	7.12 (5.58–9.07)	1.11 (1.04–1.18)	4.73 (4.03–5.55)
Bladder (squamous cell carcinoma)	J01EB02	Sulfamethizole	31	6.03 (3.81–9.55)	1.30 (1.15–1.47)	2.75 (2.11–3.58)
Oral cavity and oropharynx (squamous cell carcinoma)	N05BA02	Chlordiazepoxide	204	5.32 (4.46–6.36)	0.98 (0.93–1.02)	6.07 (5.42–6.81)
Brain and meninges (other and unspecified)	N03AX14	Levetiracetam	28	5.15 (3.24–8.18)	1.03 (0.88–1.20)	5.13 (2.62–10.04)
Larynx and hypopharynx (squamous cell carcinoma)	N07BB01	Disulfiram	119	4.99 (3.97–6.27)	1.02 (0.95–1.08)	4.81 (4.27–5.42)
Oesophagus (squamous cell carcinoma)	N05BA02	Chlordiazepoxide	49	4.69 (3.31–6.66)	0.94 (0.86–1.03)	5.36 (4.35–6.59)
Lung and trachea (adenocarcinoma)	N07BA03	Varenicline	46	4.40 (3.09–6.27)	1.08 (1.00–1.16)	3.22 (2.96–3.49)
**10 highest ORs for each doubling of cumulative dose in users**						
Bladder (squamous cell carcinoma)	J01CA08	Pivmecillinam	40	15.35 (9.40–25.06)	1.68 (1.47–1.92)	2.43 (1.81–3.25)
Bladder (squamous cell carcinoma)	J01XE01	Nitrofurantoin	30	13.58 (7.85–23.51)	1.46 (1.25–1.71)	3.87 (2.82–5.30)
Liver (Hepatocellular carcinoma)	N07BC02	Methadone	62	12.02 (7.76–18.63)	1.43 (1.26–1.62)	1.69 (0.74–3.85)
Lung and trachea (adenocarcinoma)	H05AA02	Teriparatide	46	1.52 (1.11–2.08)	1.38 (1.01–1.89)	0.32 (0.04–2.40)
Thyroidea (carcinoma, other and unspecified)	C09AA02	Enalapril	25	1.70 (1.07–2.68)	1.38 (1.12–1.70)	0.22 (0.05–0.90)
Lymphoma, non-Hodgkin	N05AH02	Clozapine	32	2.88 (1.94–4.28)	1.38 (1.16–1.64)	0.78 (0.24–2.52)
Corpus uteri (adenocarcinoma, type I)	G03CX01	Tibolone	256	3.36 (2.91–3.88)	1.37 (1.28–1.47)	1.20 (0.90–1.61)
Pancreas (adenocarcinoma)	C07AA03	Pindolol	26	1.46 (0.97–2.21)	1.32 (1.08–1.61)	0.36 (0.11–1.14)
Skin (melanoma)	C09DB01	Valsartan and amlodipine	37	1.81 (1.27–2.57)	1.32 (1.06–1.64)	0.82 (0.40–1.68)
Ovary (serous carcinoma)	M05BA01	Etidronic acid	44	1.15 (0.84–1.58)	1.31 (1.09–1.59)	0.41 (0.18–0.93)

Abbreviation: ATC, Anatomical Therapeutic Chemical classification.

### Manually Reviewed Associations

**FIGURE 1:**
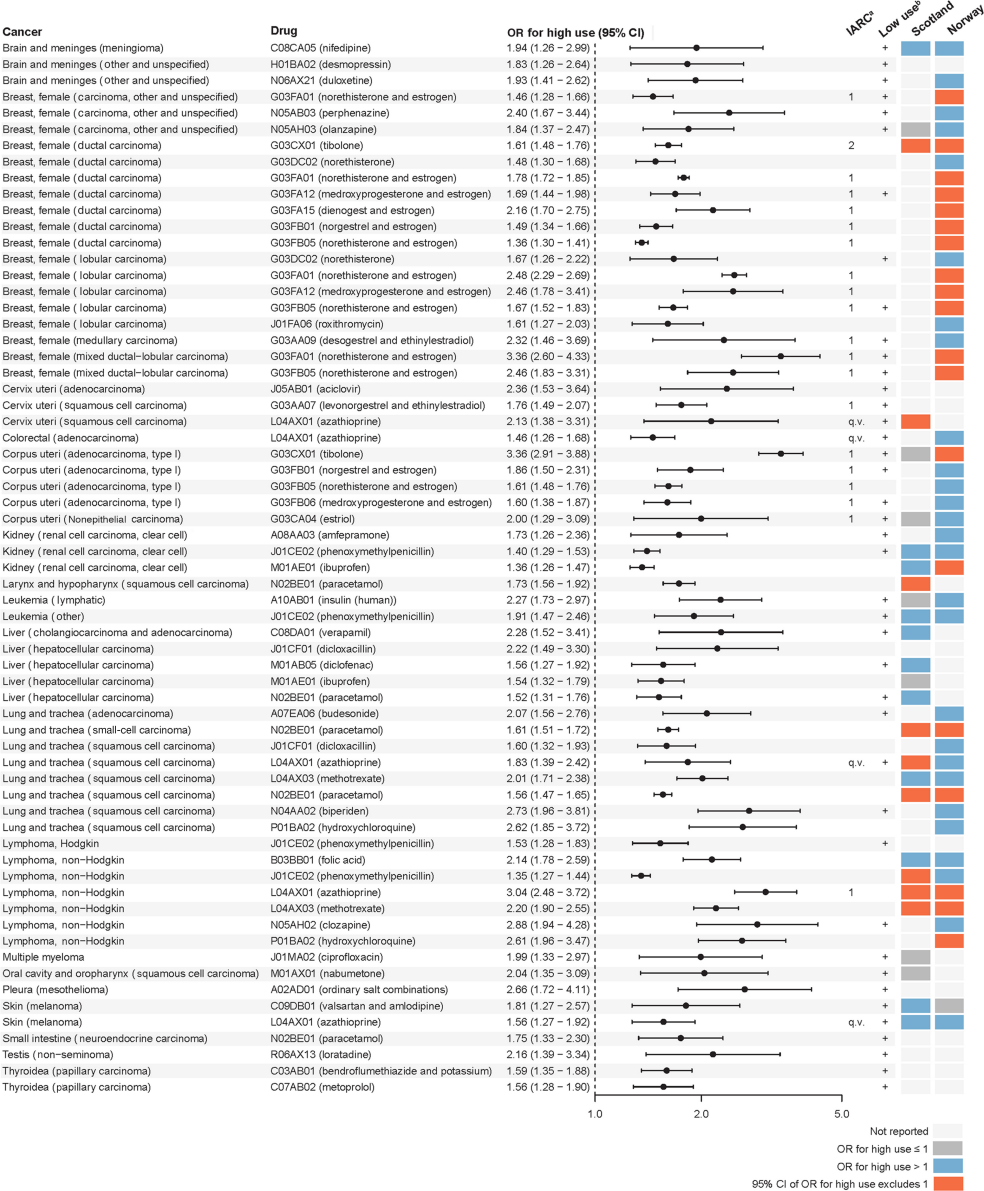
Drug–cancer associations not readily attributable to bias. The figure shows drug–cancer associations not readily explained by bias and the corresponding OR for high use (8+ prescriptions compared to never-use), the International Agency for Research on Cancer (IARC) classification of the drug–cancer association, whether the association with low-use (1–2 prescriptions compared with never-use) was neutral, and whether the association was present in the screening studies from Norway (8+ prescriptions compared with never-use) and Scotland (6+ prescriptions compared with <6 prescriptions). ^a^International Agency for Research on Cancer classification: 1, carcinogenic agents with sufficient evidence in humans; 2, carcinogenic agents with limited evidence in humans. ^b^Drug–cancer associations with a neutral association for low-use (i.e., the 95% CI includes 1) are marked with “+”. Note (q.v.): azathioprine is classified as a human carcinogen; however, only squamous cell carcinomas of the skin and non-Hodgkin lymphoma are identified as site-specific cancers with sufficient evidence in humans.

For the drug–cancer pairs in the main analyses on individual drugs and histologic subtypes of cancer (*n* = 8,373), we identified associations with an expected higher likelihood of carcinogenic drug effects based on the strength of association with high use. After this first step, 460 drug–cancer pairs remained. When additionally requiring evidence of a cumulative dose–response relationship within users of the drug, 274 drug–cancer pairs remained. These associations were manually reviewed and classified as (i) likely explained by bias (*n* = 199), (ii) implausible due to the pharmacologic properties of the drug (*n* = 10), and (iii) not readily attributable to bias (*n* = 65). Of the 65 associations classified in group (iii), 19 were classified as human carcinogens with sufficient evidence and 1 was classified as a human carcinogen with limited evidence by the IARC (2). The remaining 45 associations were not classified or not classifiable as to their carcinogenicity by the IARC. Figure 1 shows the 65 associations and the ORs for high use, the IARC classification, whether the association was neutral for low-use (1–2 prescriptions), and whether the association was present in the screening studies from Norway and Scotland (23, 24). As seen in Figure 1, the majority of drugs that were already established as carcinogenic comprised hormone replacement therapy that is classified as a cause of breast cancer and uterine cancer. Three of the 65 drug–cancer associations in group (iii) were present in all three screening studies while not being classified by the IARC with regards to carcinogenicity (small-cell carcinoma and squamous cell carcinoma of the lung and paracetamol and non-Hodgkin lymphoma and methotrexate). The rationale for the classification, the potential for bias, and selected existing literature for all 274 associations are shown in the online tool (pharmacoepi.sdu.dk/cancerscreening).

## Discussion

This study presents a hypothesis-free or agnostic approach to screen drugs for carcinogenic effects and is intended to assist in the ongoing monitoring of drug safety. All results are available online and only a few drug–cancer associations are discussed in this paper in accordance with the main aims of this study, that is, to provide a tool that may be used for hypothesis generation and as an explorative tool to inform future drug-cancer studies. On the basis of thresholds related to the strength of association with high use and a cumulative dose–response pattern, we identified 274 associations with a higher expected likelihood of representing carcinogenic effects. Because the effect estimates were prone to bias given the agnostic nature of the study, we reviewed these associations manually. This plausibility check was based on subject matter knowledge and was not readily automatized due to the multiple and varying sources of bias. The predefined thresholds to select associations for manual review were arbitrarily defined and have likely resulted in relevant associations being dropped. Acknowledging that the threshold definition is not universally applicable, we present all results online to allow researchers to explore all drug–cancer associations and to explore selected associations based on customized thresholds.

Approximately three quarters of the 274 manually reviewed associations were classified as implausible or likely explained by bias. For example, the strongest associations were observed for squamous cell carcinoma of the bladder and several antibiotics used in urinary tract infections; however, these associations likely reflect a carcinogenic effect of the underlying infection and inflammation rather than an effect of the drug itself (29). The remaining 65 associations included 20 drug–cancer pairs that were classified as carcinogenic with sufficient or limited evidence in humans by the IARC. For example, azathioprine was associated with non-Hodgkin lymphoma with an OR of 3.04 (95% CI, 2.48–3.72) an association that was present in both the Norwegian and Scottish screening study (23, 24) and consistent with the IARC classification of azathioprine as carcinogen with sufficient evidence for non-Hodgkin lymphoma in humans (30). The carcinogenic potential of azathioprine with regards to other cancers is less well established. We observed a strong association between high use of azathioprine and squamous cell carcinoma of the cervix (OR, 2.13; 95% CI, 1.38–3.31) with evidence of a cumulative dose–response pattern (OR, 1.27; 95% CI, 1.07–1.52), and a similar finding in the Scottish study (OR, 2.88; 95% CI, 1.14–7.28; ref. 24). Norwegian estimates were not available because cervical cancer was not included as an outcome in this study (23). A previous study reported a HR of 2.2 (95% CI, 1.2–3.9) for high cumulative doses of azathioprine compared with nonuse in women with autoimmune diseases (31). In this screening study, azathioprine use was also associated with colorectal adenocarcinoma, squamous cell carcinoma of the lung, and malignant melanoma. Recognizing the known carcinogenic properties of azathioprine, these observations deserve further scrutiny. Another drug–cancer association that may be worth further scrutiny was nifedipine and meningioma of the brain (OR, 1.94; 95% CI, 1.26–2.99). This association was less consistent in the two other screening studies; however, associations were not available for meningioma specifically in these studies. Confounding by indication should be addressed in future studies because hypertension is associated with increased risk of brain tumors, especially meningiomas (32).

The associations observed in this and other screening studies can be due to a true causal relation, either known or unknown, bias, or chance (33). Several complementary aspects can be used to assess the validity of each association including cumulative dose–response relationships, the specificity of the association, the risk of unmeasured or unknown confounders, and biological plausibility (34). It is considered pharmacologically plausible that cancer risk increases with higher cumulative exposures, that is, existence of a cumulative dose–response relationship (33). Conversely, if cancer risk does not increase with cumulative exposure, this may indicate that the association is non-causal and associations that are explained by reverse causation may even show inverse cumulative dose–response patterns as observed for, for example, mirabegron and prostate and bladder cancer (35). Because we estimated the OR associated with each doubling of cumulative dose within users, the lack of comparability between drug-users and never-users were mitigated, reducing the risk of confounding in these analyses. The low-use category (1–2 prescriptions) may indicate bias by, for example, confounding or selection bias because cumulative doses this low are generally unlikely to influence cancer development. The specificity of the association can be considered in relation to the exposure and outcome by comparing associations across drugs and cancers, respectively. For example, we observed that several antibiotics with different mechanisms of action were associated with adenocarcinoma of the lung and this lack of exposure specificity indicates that the observed associations are likely biased by confounding and surveillance bias. Lack of outcome specificity, that is, a given drug associated with several different cancers, may also indicate bias if the drug in question does not act as a universal carcinogen that increases the risk of several cancers. This assumption may often be reasonable considering the evidence from premarketing safety assessment of newer drugs that include *in vitro* and animal studies of carcinogenicity and the existing evidence from IARC regarding established carcinogenic effects of drugs which in general is restricted to a limited number of specific cancer sites (i.e., 1–3 sites; refs. 30, 36). Cumulative dose–response patterns and specificity of the association can be assessed directly from the study results; however, assessment of confounding and biological plausibility must incorporate external knowledge and is not readily implemented in automatic signal processing. Rather, such evaluation must be made on an individual basis for each drug–cancer association. For example, drugs associated with smoking (e.g., opioids, benzodiazepines, drugs used in chronic obstructive pulmonary disease, and varenicline) were strongly associated with most lung cancer subtypes. As an example of biological implausibility, we observed an increased OR for meningioma associated with topical corticosteroids for treatment of hemorrhoids with a cumulative dose–response relationship. However, topical corticosteroids for this indication are not absorbed to a degree that plausibly influence tumor development and the association is more likely explained by increased health care contact and surveillance bias. Such judgements require an individual assessment of drug–cancer associations of interest and relies on the researcher's subject matter knowledge. We acknowledge the subjectivity of such assessments, but we argue that statistical inference cannot be used alone to judge whether the associations may reflect causality. Another important part of the evaluation of a given drug–cancer association includes examining whether the association is replicated in other populations (37). We compared associations that were classified as not readily explained by bias with two recent screening studies from Norway and Scotland (23, 24). Online access to the Norwegian results is available at pharmacoepi.shinyapps.io/drugwas.

The methodology applied in this study is similar to existing drug–cancer screening studies. In a case–control study nested in a cohort of subscribers to the Kaiser Permanente Medical Care Program, a combination of an algorithmic approach and individual ascertainment of each association was used (38). First, associations with an OR above 1.5 with a significance of 0.01 and a dose–response pattern was kept. These associations were then reviewed for likely confounding based on clinical judgement and associations that were not likely to be explained by confounding were presented in the manuscript. The study from Scotland highlighted associations with an adjusted OR above 1.25 significant on the 1% level, and with evidence of a dose–response association (24) and the study from Norway highlighted associations based on adjusted effect estimates that were significant at the 5% level after Bonferroni correction of multiple testing. A dose–response analysis was then used to classify associations as dose-dependent or independent (23). Hypothesis-free screening of adverse effects of drugs has potential to supplement traditional pharmacovigilance systems based on spontaneous reporting of adverse drug events. Spontaneous reporting of adverse events has several limitations including underreporting of common and, as for cancers, delayed adverse events, influence of media attention, and inability to quantify risks (39, 40). However, because useful alternatives are absent, most regulatory decisions are currently based on spontaneous adverse event reports (41). Studies such as these may be implemented as an active part of the ongoing drug surveillance and thus serve in regulatory decision making.

Because we examined 13,577 associations for individual drugs, approximately 680 associations would be positive due to chance alone based on the traditional 5% significance level. The number of false positives could be reduced by adjusting for multiple testing. However, this would also reduce the likelihood of identifying associations that were due to a carcinogenic effect of the drug. Considering the exploratory and hypothesis-generating nature of our study, we preferred not to reject associations before they were subject to further evaluation that, as stated previously, cannot be made on statistical inference alone (42). Our main exposure of interest was high cumulative use defined as 8 or more filled prescriptions. It was outside the scope of this study to examine how timing of exposure was associated with cancer risk. Follow-up studies investigating individual drug–cancer associations should examine dose–response associations in more detail, for example, using flexible methods based on restricted cubic splines, examine measures of duration of use, and the impact of timing of exposure in relation to cancer risk.

Data on smoking, alcohol intake, and obesity were not available and, because they are important causes of several cancers, they may confound many of the examined drug–cancer associations (43). We adjusted for highest achieved education as a proxy of socioeconomic status but residual confounding by lifestyle and other factors must be expected. Furthermore, our screening study did not allow confounder adjustment tailored to the specific drug and cancer under scrutiny. Thus, the study should be considered hypothesis-generating and a drug–cancer association should not be interpreted causally nor used to inform clinical practice. However, associations of interest should be pursued in future studies tailored to the specific drug–cancer association under study (33, 37) based on pharmacoepidemiologic principles for establishing causal inference (44).

## Conclusions

In conclusion, hypothesis-free screening studies are feasible and may serve as useful tools in pharmacovigilance. We provide a screening tool for drug carcinogenicity aimed at hypothesis-generation and explorative purposes. Considering the hypothesis-generating nature of this work, the reported associations should be interpreted with caution and need confirmation in future studies. Hence, the results reported in this study should not be used to inform clinical decisions.

## Supplementary Material

Supplementary Data S1 Online Supplementary Material for publication containing additional information on covariate definitions (table S1 - S2) and statistical methods (methods S3) as well as the number of cases and examined drugs for each cancer under examination (table S4).
