# Supplementary material for: Identification of Drug–Cancer Associations: A Nationwide Screening Study
Source: Cancer Res Commun. 2022 Jun 29;2(6):552–60. doi: 10.1158/2767-9764.CRC-22-0026 (PMC10010324; doi:10.1158/2767-9764.CRC-22-0026)
Supplement: Supplementary Data S1 — Online Supplementary Material for publication containing additional information on covariate definitions (table S1 - S2) and statistical methods (methods S3) as well as the number of cases and examined drugs for each cancer under examination (table S4). [file crc-22-0026-s01.pdf]

ONLINE SUPPLEMENTARY MATERIAL

Identification of drug-cancer associations: A nationwide screening study

Kristensen et al

Supplementary table s1: Cancer definitions

Supplementary table s2: Definitions of covariates

Supplementary methods s3: Log_2 transformation in cumulative dose-response analyses

Supplementary table s4: Number of cases and number of evaluated drug-cancer pairs for individual drugs and drug classes

Supplementary table s1: Cancer definitions

Cancer and histologic subtypes	ICD-0-3-codes
**Bladder (ICD-10: C67)**	
Adenocarcinoma	81403 82303 82463 82603 83103 84803 84903 85603
Carcinoma, other and unspecified	80103 80123 80203 80213 80333 80413 80463
Squamous cell carcinoma	80703 80713 80723 80763 80833 80843
Urothelial carcinoma	81203 81213 81223 81303 81313
**Bones, joints and cartilage (ICD-10: C40, C41)**	
Chondrosarcoma	92203 92313 92403 92423 92433
Osteosarcoma	91803 91813 91823 91833 91853 91873 91923 91933
Sarcoma, other and unspecified	88003 88013 88043 88053 88103 88113 88153 88303 88903 91203 92503 92603 92613 93103 93703
**Brain and meninges (ICD-10: C70, C71, D32, D330-D332, D42, D430-D432)**	
Glioma	93803 93813 93823 93831 93841 93911 93913 93923 93933 93941 94003 94013 94103 94113 94121 94203 94211 94243 94253 94303 94403 94413 94423 94441 94501 94503 94513
Meningioma	95300 95301 95303 95310 95320 95323 95330 95340 95350 95370 95383 95391 95393
Other and unspecified	No histological verification required
**Breast, female (ICD-10: C50)**	
Adenocarcinoma, other	81403 81413 82003 82013 82113 82303 82403 82463 82603 83103 83153 84013 84803 84813 84903 85503 85603 85703 85723 85733 85743 85753
Carcinoma, other and unspecified	80103 80123 80133 80203 80213 80223 80323 80413 80463 80503 80703 80713 84413 85023 85303 89823
Ductal carcinoma	85003 85013 85033 85043 85073 85093 85213
Lobular carcinoma	85203
Medullary carcinoma	85103
Mixed ductal-lobular carcinoma	85223
Paget disease	85403 85413
**Breast, male (ICD-10: C50)**	
Adenocarcinoma	81403 82003 82013 82113 82463 82603 84013 84803 85003 85033 85043 85203 85403
Carcinoma, other and unspecified	80103 80463 80503 80703
**Cervix uteri (ICD-10: C53)**	
Adenocarcinoma	81403 81433 82103 82603 82623 83103 83803 83843 84303 84413 84703 84803 84813 84903 85603 85703
Squamous cell carcinoma	80523 80703 80713 80723 80733 80743 80753 80763 80833 80843
**Colorectal (ICD-10: C18-C20)**	
Adenocarcinoma	81403 81413 81433 81443 81453 82013 82103 82113 82133 82203 82303 82603 82613 82623 82633 83103 83233 84413 84603 84613 84703 84803 84813 84903 85103 85503 85603 85713 85743
Carcinoma, other and unspecified	80003 80013 80103 80123 80203 80213 80223 80333 80413 80443 80453 80463 80513 80703 80713 80833 81233
Neuroendocrine carcinoma	80133 82403 82433 82443 82463 82493
**Corpus uteri (ICD-10: C54, C55)**	
Adenocarcinoma, other	82603 83103 83233 83813 84303 84403 84413 84603 84613 84803 84903
Adenocarcinoma, type I	81403 81433 82103 82303 83803 83843 84703 85603 85703 85723 85733
Epithelial carcinoma, other and unspecified	80103 80203 80513 80703 80713 80723 80763 80833
Non-epithelial carcinoma	88003 88053 88583 88903 88913 88963 89003 89013 89103 89213 89303 89313 89333 89353 89503 89803
**Gallbladder and biliary tract (ICD-10: C23-C24)**	
Adenocarcinoma and cholangiocarcinoma	80103 80503 81403 81433 81443 81603 81623 81633 82103 82113 82303 82603 83103 84413 84703 84803 84813 84903 85603
**Kidney (ICD-10: C64)**	
Renal cell carcinoma, clear cell	83103 83123 83133 83233
Renal cell carcinoma, other and unspecified	80103 81403 82113 82903 83163 83173 83193
Renal cell carcinoma, papillary	82603

Cancer and histologic subtypes	ICD-0-3-codes
**Larynx and hypopharynx (ICD-10: C12, C13, C32)**	
Squamous cell carcinoma	80323 80513 80523 80703 80713 80723 80743 80763 80823 80833 85603
**Leukemia (ICD-10: C91-95)**	
Lymphatic (ICD-10: C91)	No histological verification required
Myeloid (ICD-10: C92)	No histological verification required
Other (ICD-10: C93-C95)	No histological verification required
**Liver (ICD-10: C22)**	
Cholangiocarcinoma and adenocarcinoma	81403 81433 81603 81623 82103 82603 83103 84403 84703 84803 84813
Hepatocellular carcinoma	81703 81713 81723 81803
**Lung and trachea (ICD-10: C33, C34)**	
Adenocarcinoma	81403 82303 82403 82493 82503 82513 82523 82533 82543 82603 82903 83233 84803 84813 84903 85503 85603
Carcinoma, other and unspecified	80013 80103 80133 80203 80213 80333 80463 81233 82003 82463 83103 84303 84703 85623 85743 89803 89823
Large cell carcinoma	80123 80143 80223 80313
Small cell carcinoma	80023 80413 80423 80433 80443 80453
Squamous cell carcinoma	80323 80523 80703 80713 80723 80733 80743 80763 80823 80833
**Lymphoma, Hodgkin (ICD-10: C81 or ICD-O-3: 965(0-9)3, 966(0-9)3)**	
**Lymphoma, Non-Hodgkin (ICD-10: C82-C86, C884 or ICD-O-3: 959(0-9)3, 967(0-9)3, 968(0-9)3, 969(0-9)3, 970(0-9)3, 971(0-9)3, 972(0-9)3)**	
**Multiple myeloma (ICD-10: C90 or ICD-O-3: 973(1-4)3)**	
**Nasal cavity and sinuses (ICD-10: C300, C31)**	
Carcinoma, other and unspecified	80103 80133 80203 80213 80413 80463 81213 81233 81403 81443 81473 82003 82303 82403 82463 82603 84303 84803 85603 85623
Squamous cell carcinoma	80323 80513 80523 80703 80713 80723 80743 80753 80763 80823 80833
**Nasopharynx (ICD-10: C11)**	
Carcinoma	80103 80113 80203 80213 80413 80463 80523 80703 80713 80723 80743 80823 80833 81233 81403 82003 84303 85253
**Oesophagus (ICD-10: C15)**	
Adenocarcinoma	81403 81433 81443 82103 82113 82303 82603 82633 83103 84303 84413 84803 84813 84903 85603
Carcinoma, other and unspecified	80103 80133 80203 80413 80463 81453 82403 82443 82463
Squamous cell carcinoma	80333 80513 80703 80713 80723 80743 80763 80833 81233
**Oral cavity and oropharynx (ICD-10: C01-C06, C09, C10, C14)**	
Carcinoma, other and unspecified	80003 80013 80103 80203 80213 80413 80463 81233 81403 81473 82003 82303 82463 82603 83103 84303 84413 84503 84703 84803 85253 85503 85603 85623
Squamous cell carcinoma	80323 80513 80523 80703 80713 80723 80743 80753 80763 80823 80833
**Ovary (ICD-10: C56)**	
Clear cell carcinoma	83103 83133 84903
Endometroid carcinoma	83803 83813 85703 89333
Epitehelial carcinoma, other and unspecified	80103 80123 80133 80203 80213 80333 80413 80423 80463 80503 80703 80713 81303 81403 81413 82003 82303 82463 82603 83233 84403 84503 85603 85713 89343 89803 90003 90143
Mucinous carcinoma	84703 84713 84743 84803 84813 90153
Non-epithelial neoplasms	82403 82433 82493 86203 86313 88003 88103 88903 89313 89353 89503 89513 90603 90643 90713 90803 90843 90853 91003
Serous carcinoma	84413 84603 84613
**Pancreas (ICD-10: C25)**	
Adenocarcinoma	81403 81433 81453 82113 82303 82603 83103 84303 84403 84413 84523 84533 84703 84713 84803 84813 84903 85003 85033 85503 85513 85603
Carcinoma, other and unspecified	80003 80013 80103 80123 80203 80213 80223 80313 80333 80353 80413 80463 80703 80713 80723 80833
Neuroendocrine carcinoma	80133 81503 81513 81523 82403 82433 82443 82463
**Pleura (ICD-10: C384, C450)**	
Mesothelioma	90503 90513 90523 90533

Cancer and histologic subtypes	ICD-0-3-codes
**Prostate (ICD-10: C61)**	
Adenocarcinoma	81403 81413 82113 82303 82603 83103 83303 84803 84813 84903 85003 85033 85503
Carcinoma, other and unspecified	80103 80133 80203 80213 80223 80413 80423 80463 80703 81203 81303 81313 82013 82403 82463 83153 85603 85713 85723 85743
**Renal pelvis and ureter (ICD-10: C65, C66)**	
Urothelial carcinoma	81203 81213 81223 81303 81313
**Salivary glands (ICD-10: C07, C08)**	
Malignant tumours	80013 80103 80123 80133 80203 80213 80413 80453 80823 80833 81403 81473 82003 82013 82303 82463 82603 82903 83103 84303 84403 84503 85003 85253 85503 85623 89403 89823
**Skin (ICD-10: C43)**	
Melanoma	87203 87213 87303 87403 87413 87423 87433 87443 87453 87603 87613 87723 87803
**Small intestine (ICD-10: C17)**	
Adenocarcinoma	81403 81443 81453 82103 82303 82603 82613 83103 84603 84803 84813 84903
Neuroendocrine carcinoma	82403 82413 82433 82443 82463 82493
**Soft tissue (ICD-10: C46-C49 or ICD-O-3: 89363)**	
Fibrosarcoma	88103 88113 88303
GIST	89363
Liposarcoma	88503 88513 88523 88533 88543 88553 88583
Myosarcoma	88903 88913 88953 88963 89003 89013 89023 89103 89203
Sarcoma, other and unspecified	88003 88013 88023 88033 88043 88053 88403 89353 90403 90413 90423 90433 90443 91203 91303 91403 91503 91703 92603 95403 95603 95713 95803 95813
**Stomach (ICD-10: C16)**	
Adenocarcinoma	81403 81433 81443 81453 82103 82113 82303 82603 83103 84403 84413 84803 84813 84903 85603
Carcinoma, other and unspecified	80103 80123 80203 80413 80703 80713 80823 80833
Neuroendocrine carcinoma	80133 81533 82403 82443 82463
**Testis (ICD-10: C62)**	
Non-seminoma	90643 90653 90703 90713 90723 90803 90813 90843 90853 91003 91013
Seminoma	90613 90623 90633
**Thyroidea (ICD-10: C73)**	
Anaplastic carcinoma	80203 80213 80313
Carcinoma, other and unspecified	80103 80703 81403 82303 82903 85103
Follicular carcinoma	83303 83313 83323 83353
Papillary carcinoma	80503 82603 83403 83413 83423 83433

Supplementary table s2: Definitions of covariates

Charlson comorbidity index entity.*	ICD-10	Weight
Myocardial infarction	I21.x, I22.x, I25.2	1
Congestive heart failure	I09.9, I11.0, I13.0, I13.2, I25.5, I42.0, I42.5–I42.9, I43.x, I50.x, P29.0	1
Peripheral vascular disease	I70.x, I71.x, I73.1, I73.8, I73.9, I77.1, I79.0, I79.2, K55.1, K55.8, K55.9, Z95.8, Z95.9	1
Cerebrovascular disease	G45.x, G46.x, H34.0, I60.x–I69.x	1
Dementia	F00.x–F03.x, F05.1, G30.x, G31.1	1
Chronic pulmonary disease	I27.8, I27.9, J40.x–J47.x, J60.x–J67.x, J68.4, J70.1, J70.3	1
Rheumatic disease	M05.x, M06.x, M31.5, M32.x–M34.x, M35.1, M35.3, M36.0	1
Peptic ulcer disease	K25.x–K28.x	1
Mild liver disease	B18.x, K70.0–K70.3, K70.9, K71.3–K71.5, K71.7, K73.x, K74.x, K76.0, K76.2–K76.4, K76.8, K76.9, Z94.4	1
Diabetes without chronic complication	E10.0, E10.1, E10.6, E10.8, E10.9, E11.0, E11.1, E11.6, E11.8, E11.9, E12.0, E12.1, E12.6, E12.8, E12.9, E13.0, E13.1, E13.6, E13.8, E13.9, E14.0, E14.1, E14.6, E14.8, E14.9	1
Diabetes with chronic complication	E10.2–E10.5, E10.7, E11.2–E11.5, E11.7, E12.2–E12.5, E12.7, E13.2–E13.5, E13.7, E14.2–E14.5, E14.7	2
Hemiplegia or paraplegia	G04.1, G11.4, G80.1, G80.2, G81.x, G82.x, G83.0–G83.4, G83.9	2
Renal disease	I12.0, I13.1, N03.2–N03.7, N05.2– N05.7, N18.x, N19.x, N25.0, Z49.0–Z49.2, Z94.0, Z99.2	2
Moderate or severe liver disease	I85.0, I85.9, I86.4, I98.2, K70.4, K71.1, K72.1, K72.9, K76.5, K76.6, K76.7	3
AIDS/HIV	B20.x–B22.x, B24.x	6

*The same comorbidities with different severity were mutually exclusive: diabetes with chronic complications and diabetes without chronic complications and mild liver disease and moderate or severe liver disease.

Education		
None or basic education	*National classification of educational programs (Uddannelsesniveau)*	10-20
High school or vocational training		30-50
Higher education		60-80
Unknown		-

Supplementary methods s3: Log_2 transformation in cumulative dose-response analyses

Cumulative dose-response patterns was analyzed using a log_2 transformation of the cumulative number of filled prescriptions. This way, the shape of the dose-response curve can be modeled flexibly without imposing requirements for the shape of the dose-response curve between cumulative dose and cancer risk. This metric does not primarily measure the association for high cumulative doses, but rather the shape of the dose-response curve according to the estimated odds ratio (OR) as visualized in the curves below. The curves show the relationship between the odds of cancer and increasing cumulative dose according to different values of the OR for the log_2-transformed cumulative dose. It is apparent that ORs above 2 are equivalent to an accelerating effect, ORs between 1 and 2 to an effect that shows some saturation and an OR of 1 to a constant cancer risk. If we can assume a monotonous dose-response association, i.e., a systematically increasing (or decreasing) effect over the entire dose range, exposure transformation by a log-scale preserves the statistical power entirely.

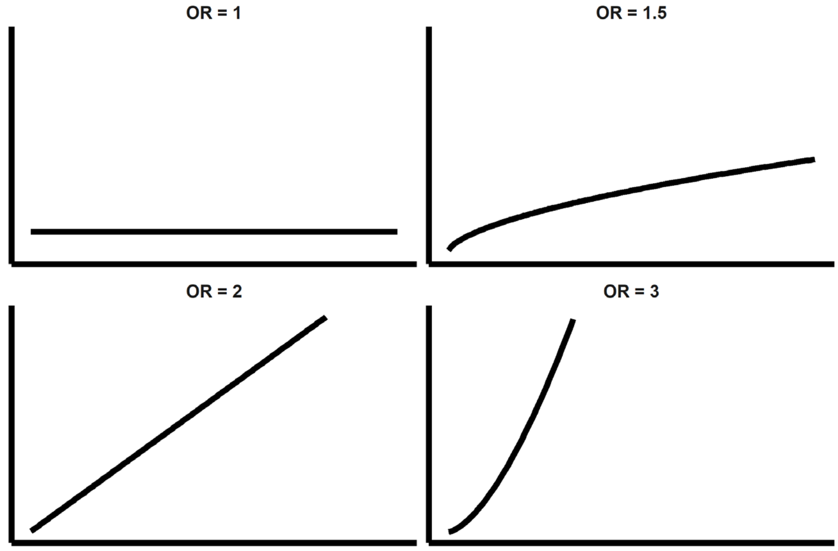

Supplementary table s4: Number of cases and number of evaluated drug-cancer pairs for individual drugs and drug classes

Cancer	Cases (n)	Drugs, 5th ATC level	Drugs, 4th ATC level
**Bladder**	13,377	284	169
Adenocarcinoma	559	11	19
Carcinoma, other and unspecified	279	6	10
Squamous cell carcinoma	413	16	21
Urothelial carcinoma	12,126	269	164
**Bones, joints and cartilage**	673	10	16
Chondrosarcoma	367	4	9
Osteosarcoma	132	0	0
Sarcoma, other and unspecified	174	0	0
**Brain and meninges**	16,001	311	189
Glioma	5728	136	103
Meningioma	3335	108	87
Other and unspecified	6938	246	152
**Breast, female**	73,589	544	271
Adenocarcinoma, other	3909	150	104
Carcinoma, other and unspecified	5439	229	143
Ductal carcinoma	54,238	486	255
Lobular carcinoma	8662	242	148
Medullary carcinoma	376	6	6
Mixed ductal-lobular carcinoma	736	28	35
Paget disease	229	2	3
**Breast, male**	476	11	20
Adenocarcinoma	448	9	16
Carcinoma, other and unspecified	28	0	0
**Cervix uteri**	5850	129	98
Adenocarcinoma	1350	36	42
Squamous cell carcinoma	4500	101	88
**Colorectal**	65,636	508	260
Adenocarcinoma	63,922	503	258
Carcinoma, other and unspecified	565	23	27
Neuroendocrine carcinoma	1149	37	44
**Corpus uteri**	11,695	285	169
Adenocarcinoma, other	1371	56	62
Adenocarcinoma, type I	9175	244	152
Epithelial carcinoma, other and unspecified	94	0	0
Non-epithelial carcinoma	1055	36	49
**Gallbladder and biliary tract**	2262	99	84
Adenocarcinoma and cholangiocarcinoma	2262	99	84
**Kidney**	9970	266	157
Renal cell carcinoma, clear cell	8720	242	147
Renal cell carcinoma, other and unspecified	658	20	33
Renal cell carcinoma, papillary	592	30	33
**Larynx and hypopharynx**	5068	155	112
Squamous cell carcinoma	5068	155	112
**Leukemia**	12,761	275	167
Lymphatic	7561	218	133
Myeloid	4357	149	107
Other	843	33	50

Cancer	Cases (n)	Drugs, 5th ATC level	Drugs, 4th ATC level
**Liver**	3331	151	112
Cholangiocarcinoma and adenocarcinoma	1081	50	59
Hepatocellular carcinoma	2250	110	92
**Lung and trachea**	58,683	502	255
Adenocarcinoma	25,944	396	211
Carcinoma, other and unspecified	9436	250	156
Large cell carcinoma	853	19	31
Small cell carcinoma	9612	270	161
Squamous cell carcinoma	12,838	305	179
**Lymphoma, Hodgkin**	2004	39	48
**Lymphoma, Non-Hodgkin**	16,947	326	189
**Multiple myeloma**	6058	195	123
**Nasal cavity and sinuses**	841	28	37
Carcinoma, other and unspecified	287	7	9
Squamous cell carcinoma	554	16	23
**Nasopharynx**	379	3	7
Carcinoma	379	3	7
**Oesophagus**	6950	207	133
Adenocarcinoma	3510	134	107
Carcinoma, other and unspecified	403	10	17
Squamous cell carcinoma	3037	100	82
**Oral cavity and oropharynx**	9617	228	140
Carcinoma, other and unspecified	497	9	18
Squamous cell carcinoma	9120	223	138
**Ovary**	7887	223	133
Clear cell carcinoma	333	3	10
Endometroid carcinoma	664	18	26
Epitehelial carcinoma, other and unspecified	1517	62	65
Mucinous carcinoma	671	14	21
Non-epithelial neoplasms	207	0	1
Serous carcinoma	4495	146	108
**Pancreas**	10,822	274	165
Adenocarcinoma	9212	256	154
Carcinoma, other and unspecified	1250	50	59
Neuroendocrine carcinoma	360	9	11
**Pleura**	1652	49	53
Mesothelioma	1652	49	53
**Prostate**	61,413	441	223
Adenocarcinoma	61,024	440	223
Carcinoma, other and unspecified	389	7	12
**Renal pelvis and ureter**	1213	46	55
Urothelial carcinoma	1213	46	55
**Salivary glands**	757	17	26
Malignant tumours	757	17	26
**Skin**	29,676	380	205
Melanoma	29,676	380	205
**Small intestine**	1354	49	59
Adenocarcinoma	709	20	32
Neuroendocrine carcinoma	645	27	35

Cancer	Cases (n)	Drugs, 5th ATC level	Drugs, 4th ATC level
**Soft tissue**	3521	116	92
Fibrosarcoma	469	11	16
GIST	926	31	43
Liposarcoma	651	11	19
Myosarcoma	522	11	20
Sarcoma, other and unspecified	953	24	29
**Stomach**	7978	226	133
Adenocarcinoma	7488	218	127
Carcinoma, other and unspecified	313	7	11
Neuroendocrine carcinoma	177	4	7
**Testis**	4702	33	40
Non-seminoma	1976	6	11
Seminoma	2726	15	27
**Thyroidea**	3685	103	91
Anaplastic carcinoma	169	3	6
Carcinoma, other and unspecified	349	9	14
Follicular carcinoma	624	15	23
Papillary carcinoma	2543	72	72
**Total**	456,828	13,577	8996
